# Operationalizing the One Health approach at the intersection of research and development: a study protocol for the ELUZO project in Senegal and Burkina Faso

**DOI:** 10.1080/16549716.2026.2705653

**Published:** 2026-07-31

**Authors:** Marine Hubert, Magali Martin, Hélène Carabin, Jane Parmley, Maude Gilbert-Vanasse, Alexis Kafando, Rosalie Seck, Heather M. Murphy, Mouhamadou Faly Ba, Abdoul Kader Ilboudo, Rianatou Bada Alambédji, Malek Batal, Abel Bicaba, Frank Lionel Bouéko Bicaba, Rosanne Blanchet, François M. Castonguay, Amadou Ibra Diallo, Fatou Diop, Thomas Druetz, Marie Jobin‑Gélinas, Asséta Kagambéga, Adama Kazienga, Sylvie Korogo, Muriel Mac-Seing, Marie‑Douce Primeau, Mylène Ratelle, Ndeye Mareme Sougou, Philomène Coumba Tine, Zékiba Tarnagda, Adama Faye, Michèle Bouchard

**Affiliations:** a Groupe de recherche en épidémiologie des zoonoses et santé publique (GREZOSP), Université de Montréal, Saint-Hyacinthe, Québec, Canada; b Faculté de médecine vétérinaire, Université de Montréal, Saint-Hyacinthe, Montréal, Québec, Canada; c Centre de recherche en santé publique (CReSP), Université de Montréal, Montréal, Québec, Canada; d UdeM international, Université de Montréal, Montréal, Québec, Canada; e École de Santé Publique, Université de Montréal, Montréal, Québec, Canada; f Ontario Veterinary College, University of Guelph, Guelph, Ontario, Canada; g Société d’étude et de Recherche en Santé Publique (SERSAP), Ouagadougou, Burkina Faso; h COMI (Cooperation for the World in Development), Roma, Italy; i Ministère de l’Agriculture, de la Souveraineté Alimentaire et de l’Élevage (MASAE), Dakar, Senegal; j Institut de Santé et Développement, Université Cheikh Anta Diop de Dakar (UCAD), Dakar, Sénégal; k Institut de Recherche en Sciences de la Santé(IRSS), Ouagadougou, Burkina Faso; l Département de Santé Publique et Environnement, École Inter-États des Sciences et Médecine Vétérinaires (EISMV), Dakar, Sénégal; m Institut des sciences de l’environnement, Université Cheikh Anta Diop de Dakar (UCAD), Dakar, Sénégal; n Unitéde santé internationale (USI), Université de Montréal, Montréal, Québec, Canada; o Université Joseph Ki-Zerbo, Ouagadougou, Burkina Faso; p Association One Health (ASSO-OHBF), Ouagadougou, Burkina Faso

**Keywords:** Zoonoses, One Health, West Africa, women empowerment, community-based interventions

## Abstract

Small-scale livestock farming remains central to food security and income-generation in several low and middle countries (LMIC), but it can also be an important source of zoonoses in areas where access to water and sanitation is poor. The role played by women in this sector is often overlooked. ELUZO is an international, intersectoral, and multidisciplinary One Health project that aims to reduce the impact of zoonotic diseases and empower women in rural communities in Senegal and Burkina Faso. The project adopts a women-centered approach, integrating development and research using a Theory of Change (ToC) framework to combine measurement tools that meet Global Affairs Canada’s requirements with scientific rigour. As part of the study protocol, we co-designed tools to collect baseline data using mixed methods to generate a comprehensive picture of the human, animal and environmental health concerns in each participating community. The baseline findings were then used to guide selection and design of interventions. The decision-making process of the project is carried out collaboratively with community members, ensuring local realities and needs are reflected. The final stage of the project will be implementation and assessment of the effectiveness of the community interventions. Additionally, the team will provide training sessions on One Health and gender equity. This paper describes the study protocol developed to guide the ELUZO project. The protocol includes the governance structure, objectives and methods used to design a gender-sensitive, community-driven and multisectoral One Health intervention project to reduce the impacts of zoonotic disease in low-resource settings.

## Paper Context

- **Main findings**: The paper describes a women-centered, community-driven framework to operationalize a One Health approach in rural Africa to fight against zoonoses.
- **Added knowledge**: The study demonstrates the importance of integrating gender equity, participatory methods and bridging research and development in community-level initiatives.
- **Global health impact for policy and action**: The paper provides a model for designing integrated One Health initiatives that engage communities, especially women, in zoonotic disease prevention.

## Background

In sub-Saharan Africa (SSA), more than 50% of the population depends on agriculture and livestock production for food and livelihood [1]. In Senegal, 44.5% of all households practice agricultural activities, 68% in rural areas, with livestock production contributing 3.8% to the national Gross Domestic Product (GDP) [2,3]. In Burkina Faso, 53.4% of households raise livestock, 69.2% in rural settings [4], and the sector accounts for 8% of national GDP [5].

Women are deeply involved in agricultural production in SSA, with 66% of all women employed in the sector. In livestock production, women are primarily responsible for small-scale management of smaller livestock animals (poultry, pigs, small ruminants) [6]. Yet, significant gender disparities persist [7]. In Senegal, women represent 48.7% of the agricultural workforce [8], but own less than 1% of agricultural land, and earn 25% less than men [9]. In Burkina Faso, 29.2% of women work in agriculture, but only 0.2% hold land rights and they face a 37.3% wage gap [5].

Zoonoses are diseases that spread between animals and humans sharing a common environment [10], with 61% of known infectious diseases of humans capable of infecting other animals [11,12]. The emergence and spread of zoonotic pathogens have been linked to close contact with animals, consumption of contaminated food or water, socio-cultural practices, such as hunting and consuming wildlife and open defecation, and a lack of meat inspection infrastructure [13,14]. Broader scale anthropogenic drivers, such as deforestation, urbanization, intensive livestock farming, increasing movement of humans and animals, human behaviour and climate change, also increase the risk of zoonotic disease emergence [15]. The 2025 Lancet One Health commission highlighted how climate change, biodiversity loss and pollution influence disease dynamics and emphasized the need to address inequities in health, food and water security, and waste management [16]. In poor communities with limited access to healthcare, as in rural Senegal and Burkina Faso, inadequate sanitary conditions and limited access to water and other resources further increase the vulnerability of humans and livestock to zoonotic risks [17,18]. Further complicating the situation, in communities where animal farming is an important source of food and income, people fear economic loss if a carcass is condemned following meat inspection or a disease is detected at the sale of live animals. This limits the implementation of regulated surveillance and slaughtering systems and subsequent reporting of diseases, especially in the absence of financial compensation [19]. A One Health approach that aims to strengthen human, animal and ecosystem health capacity is essential to effectively and sustainably tackle these interdependent and compounding challenges [20].

Burkina Faso and Senegal have applied the One Health Zoonotic Disease Prioritization (OHZDP) [21] tool to prioritize zoonoses at the national level, based on criteria such as disease incidence, severity and socio-economic impact [22,23]. However, the OHZDP tool is designed for use by government representatives, academics, and international stakeholders, not community members, potentially creating a gap between identified national priorities and community-level realities. Participatory research initiatives can help address this gap by engaging community members in data collection, decision-making processes, and the development of locally relevant interventions to improve health outcomes [24]. As such, community participation is increasingly recognized as an essential component of public health and One Health initiatives, as it enables identification of context-specific priorities, local knowledge generation, and development and implementation of community-relevant actions [25]. In addition, gender-responsive approaches are particularly important in low-resource settings where women play a central role in livestock production to ensure that interventions are relevant, contextually appropriate and empower women [26].

Recognizing these gaps, Global Affairs Canada (GAC) launched a call for proposals under the theme ‘Community-Level One Health’, encouraging multisectoral initiatives that combine academic expertise with community knowledge across multiple countries to co-create interventions that improve women’s health and empowerment. In response, the ELUZO project (‘*Elles LUttent contre les Zoonoses*’ – They (women and girls) fight against zoonoses – In French, ‘elles’ refers specifically to women and girls) [27], was co-developed to strengthen health and community resilience to the impacts of zoonoses in rural Senegal and Burkina Faso using a women-centered One Health approach.

This paper describes the ELUZO project study protocol, specifically the conceptual framework, governance structure, participatory approach and overall methodological design to address the challenges outlined above. By documenting the approach used, the authors aim to contribute to the literature on One Health implementation.

## ELUZO framework and objectives

### Overall project goal

The ELUZO project is a transdisciplinary, intersectoral and women-centered One Health initiative aiming to improve community health and empower women and girls in regions where the risk of zoonoses is high.

In this paper, the term *women* is used as an umbrella term to include adult women (aged 18 and above) and adolescent girls (aged 15–17) who participated in the project.

The project focuses on rural communities in Senegal and Burkina Faso where women are engaged in small-scale livestock farming activities, especially of pigs, small ruminants and poultry. Recognizing the importance of community participation in One Health research, the ELUZO team places particular attention on the specific needs of women and ensures their active participation in shaping priorities making decisions, co-designing and implementing interventions with them. This is also the source of the name given to the project ‘ELUZO,’ which highlights the central role of women and girls in fighting against zoonoses. The ELUZO team also seeks to promote greater awareness of gender inequities among men, community leaders, and professionals, without imposing external norms, and builds on local values, strengths, and priorities expressed by community members.

Overall, ELUZO was designed to bridge international development requirements with research rigour and scientific methods. Beyond describing a specific project, this study protocol provides an example of how to combine Theory of Change (ToC) with participatory research using mixed methods for data collection and a One Health approach. As such, it contributes to the literature on implementation of participatory research and operationalization of One Health in low-resource settings.

Given that the ELUZO project operates at the intersection of research and development, methodological and operational decisions were taken collaboratively to account for all the perspectives. Indeed, researchers tend to emphasize the representativeness and scientific quality, whereas development practitioners prioritize criteria related to vulnerability and operational feasibility. Local communities, in turn, may hold differing views about who should be selected, and thus supported, to participate in the project activities, based on their capacity to contribute and local power dynamics. As such, ELUZO followed a collaborative process involving local and international researchers, local non-governmental organization partners, and community members in planning, decision-making and implementation.

### Theory of change

A unique feature of the ELUZO project is that it combines international development components [28], guided by GAC’s results-based management (RBM) approach, with rigorous research methods, including mixed methods for data collection. Development projects typically focus on achieving predefined outcomes through structured chains of activities, while research aims to generate new knowledge by testing hypotheses. Integrating these two approaches required a framework that could align both perspectives.

To achieve this, ELUZO adopted a ToC approach, a structured method widely used to guide policies, programs and interventions by depicting causal steps that lead to desired outcomes. A ToC model provides clear pathways connecting activities to outputs, immediate results, and ultimate impacts. By depicting how project activities lead to desired outcomes, a ToC helps ensure process transparency and identifies key places and opportunities for monitoring, evaluation, and stakeholder participation throughout the project [29,30]. These practical benefits make a ToC approach particularly suitable for complex initiatives like ELUZO.

**Figure 1.**
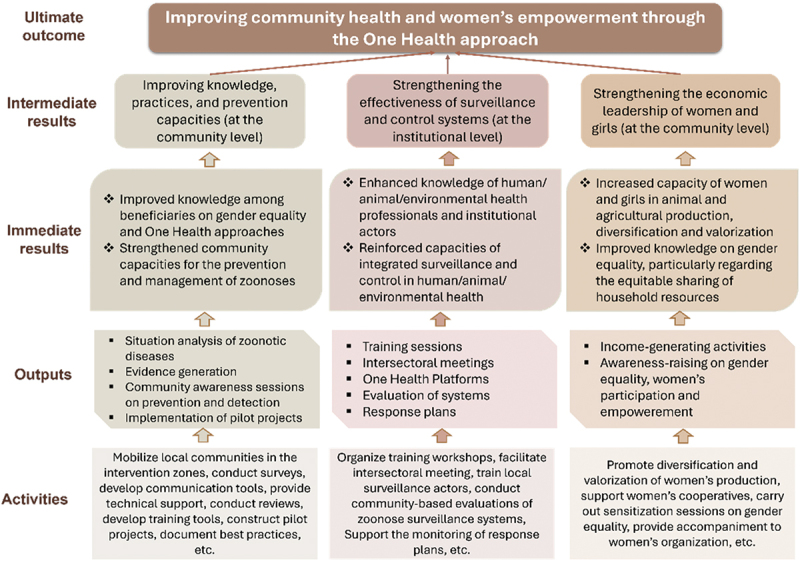
ELUZO theory of change. ELUZO encompasses approximately 70 unique activities, 19 outputs, 6 immediate results, 3 intermediate results, and one ultimate outcome, namely, to improve community health and empower women (see supplementary material for full project results chain).

The ELUZO ToC was co-developed by the research team and local partners during the project design stage, prior to start of data collection (Figure 1). Activities (e.g. surveys, focus groups) were designed to produce outputs (e.g. overview of the sanitary situation in the participating communities, selection of pilot projects and income-generating activities), which lead to immediate results (e.g. strengthened community capacity for prevention of zoonoses), three main intermediate results (e.g. strengthening the economic leadership of women and girls) and one ultimate project outcome, improving community health and empowering women using a One Health approach.

### Theory of change: project results chain

In line with a community-led initiative, the methodological steps were first informed by the needs and priorities identified by community members during field consultations.

### Research objectives

Building on the ToC, specific ELUZO research objectives were identified and refined to align with the expected outcomes. Given the central role of women and girls in small-scale livestock management and their risk of exposure to zoonotic diseases, gender equity considerations were systematically integrated throughout all project phases and objectives, rather than treated as a separate goal.

The project includes four research objectives and one capacity-building objective:

- Research objective 1: To describe the local contexts and knowledge, attitudes, and practices (KAP) that shape how small-scale livestock are raised by women and girls in rural communities of Senegal and Burkina Faso.
- Research objective 2: To co-identify and prioritize the most important animal, human and environmental health concerns in each of the participating communities with women and girls involved in livestock management and production.
- Research objective 3: To co-develop pilot interventions and revenue-generating activities with community members that address one of the top three concerns identified in research objective 2.
- Research objective 4: To evaluate the implementation and effectiveness of the interventions developed in research objective 3.
- Capacity building objective: To develop educational materials about One Health and gender equity adapted to different audiences.

As an additional cross-cutting objective, ELUZO also seeks to develop and document tools and methods to integrate research into traditional development projects, ensuring that the process informs both scientific inquiry and implementation. We consider this project to be a participatory and implementation research initiative, in which research objectives are aligned with outcomes of our results-based management approach.

## Methodological framework

### Project governance

In response to the GAC call for proposals, the ELUZO team re-invigorated established relationships among academic and institutional partners in Canada, Senegal and Burkina Faso to co‑design a joint, multi-country initiative.

ELUZO is coordinated by representatives from 11 organizations: two in Canada, four in Senegal, and five in Burkina Faso. The Université de Montréal (UdeM) [31], specifically its international affairs office called ‘UdeM international’ and its International Health Unit (‘USI’), is the leading organization responsible for the overall management and project coordination. Researchers at UdeM and the University of Guelph [32] bring expertise in zoonoses’ epidemiology, global public health, One Health, environmental health, integrated surveillance systems and water, hygiene, and sanitation (WASH) [32]. A partnership agreement defines the division of responsibilities between the two Canadian institutions and their accountability to GAC.

In Senegal and Burkina Faso, country consortium agreements were signed between UdeM and participating organizations in both countries. One partner per country was identified as the national focal point to coordinate implementation with other partners. These were ‘Cooperazione per il Mondo in Via di Sviluppo’ (COMI) [33] in Senegal and the ‘Société d’Études et de Recherche en Santé Publique’ (SERSAP) [34] in Burkina Faso. Researchers in the partner countries provide expertise in human health, veterinary health, social sciences, public and community health and One Health promotion. Detailed descriptions of partner organizations involved along with their roles are provided in Supplementary Table S1.

ELUZO established a multi-level governance structure with three main committees:

1. a steering committee including representatives of all partner organizations;
2. an operations committee made up of country coordinators, gender advisors, administrative and financial officers (who oversee administrative and financial management) with a Canadian lead;
3. a scientific committee including scientific co-directors from each country and a Canadian scientific coordinator.

In addition, co-investigators with thematic expertise act as resource personnel and are consulted on specific issues. This model promotes transparent decision-making and equitable participation of partners in Canada, Senegal, and Burkina Faso.

### Governance structure of ELUZO

**Figure 2.**
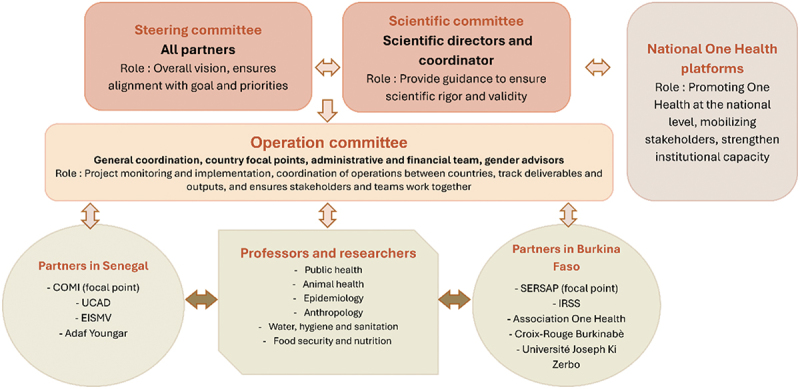
Governance structure of the ELUZO project, including steering, scientific and operation committee, partners in all countries and their roles.

Beyond coordination and accountability, the governance structure depicted in Figure 2 illustrates how One Health can be operationalized by connecting experts from multiple disciplines and countries. Communities are directly involved in decision-making, ensuring that field realities inform the entire process while bridging research and development objectives.

### Collaboration with national One Health platforms and ministries

Collaboration with national stakeholders was part of the study design and implementation strategy. Indeed, national One Health platforms and ministries in both countries facilitated access to regional authorities, which in turn helped identify eligible communities. They also provided official surveillance documents, participated in the qualitative data collection components of ELUZO, and were actively involved in the creation and validation of training modules and tools on the One Health approach.

At the community level, the ELUZO team worked with human, animal, and environmental health agents who usually carry out activities mandated by their respective ministries and transmit separate reports to their regional and then national-level representatives, with little communication, coordination, or cooperation across sectors. ELUZO brought these agents into unified ‘One Health units’ with the support of the ministries involved. Within each community, these One Health units function to facilitate implementation of interventions, coordinate data collection at the human-animal interface and serve as key actors in the training and awareness-raising sessions.

### Study design

In line with the objectives outlined above, the project was implemented as four main activities;

1. Baseline assessment: This step used mixed methods (quantitative surveys, qualitative interviews, focus groups, participatory walks and literature reviews) to gather data and generate a global overview of the ‘One Health’ situation in the participating communities, including health and economic realities, livestock diversity, gender-based dynamics, available resources and surveillance methods. This addressed research objective 1.
2. Data triangulation: All data from the quantitative and qualitative components of the baseline assessment, along with the literature reviews were combined (triangulated) for each village to identify major health (animal, human and environmental) challenges and contextual factors shaping the communities. This process contributes to research objective 2.
3. Intervention and implementation: Based on the data triangulation results, pilot projects and income generating activities were identified and prioritized collaboratively with the communities and implemented through a participatory process. The data collected also informed the content of awareness and training sessions on One Health and gender equality, supporting the project’s capacity building objective. These activities respond to research objective 3.

**Figure 3.**
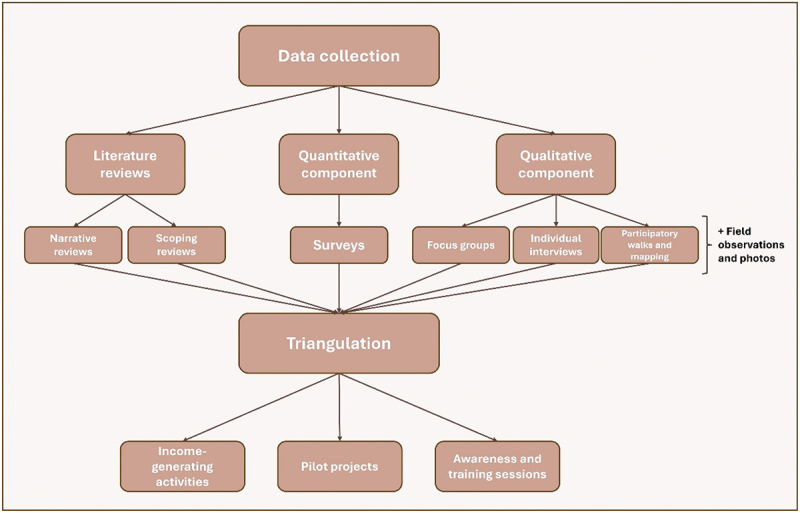
Overview of the study design, including data collection components (literature reviews, quantitative surveys, qualitative methods), triangulation, and intervention development.

All these steps are depicted in Figure 3.

### Study design and methodological framework

(4) Monitoring and evaluation: Follow-up and evaluation of activities included in the ToC diagram (Figure 1) are integrated throughout all project activities. This provides an ability to assess the impact of the interventions and contributes to research objective 4.

ELUZO is designed as a contextually adapted pre-post intervention study comparing conditions before and after the implementation of community-driven activities. All project activities are planned within a five-year period from 2023 to 2028.

### Selection of villages

**Figure 4.**
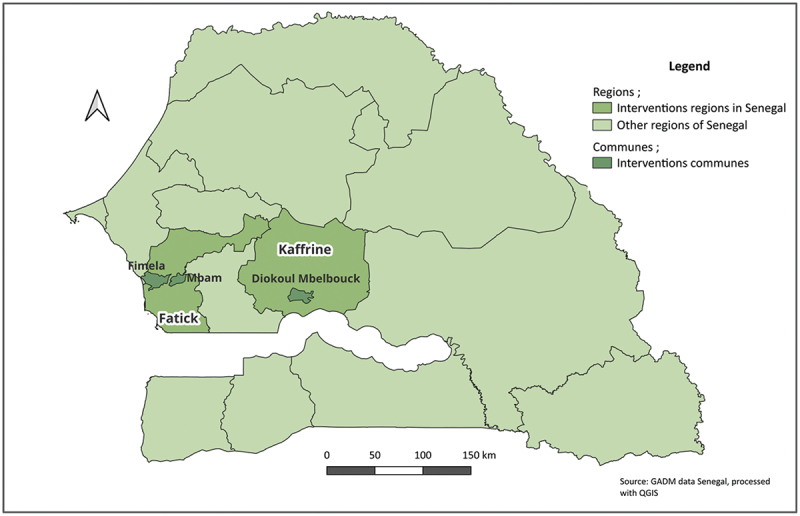
Map of ELUZO intervention regions and communes in Senegal.

**Figure 5.**
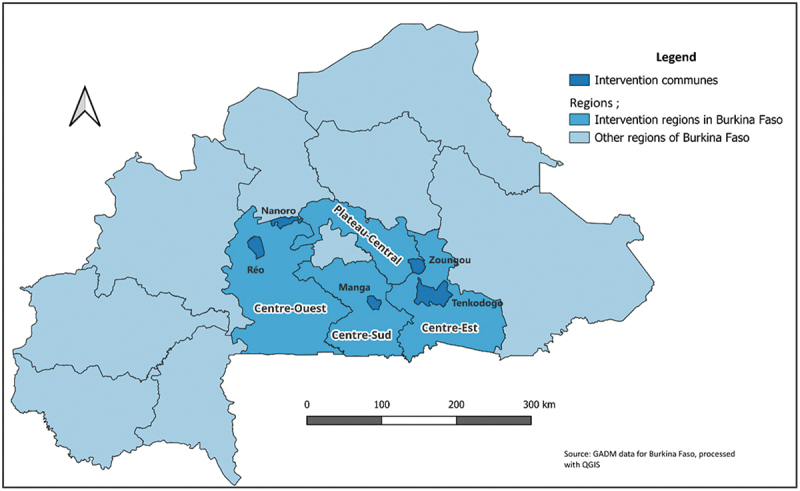
Map of ELUZO intervention regions and communes in Burkina Faso.

Selection of the participating villages started with identifying departments or communes in each country where local ELUZO partners were established, which are defined as the area of intervention. In Senegal, these were the communes of Mbam, Diokoul Mbelbouck and Fimela (Figure 4). In Burkina Faso, the communes of Tenkodogo, Zoungou, Manga, Reo and Nanoro were selected (Figure 5).

All eligible villages were then identified according to pre-identified criteria established by the research team (Table 1). Village eligibility was assessed through available census data, knowledge of local research team members and preliminary meetings conducted by local partners with the villages authorities to confirm their interest and consent to engage.

**Table 1.** Eligibility criteria for the selection of villages in Burkina Faso and Senegal.

Criteria 1	Accessibility and safety: Villages must be easily accessible and safe, allowing project facilitators to work with minimal risk.
Criteria 2	Minimum village size: Villages needed a minimum number of inhabitants (approximately 1,000 persons).
Criteria 3	Livestock farming by women: Villages must include women engaged in small livestock farming, including at least two of the following species: poultry, pigs, or goats/sheep.
Criteria 4	Vulnerability: Priority was given to villages with few prior interventions, in rural areas far from large urban centres, including those with a high concentration of poor women practicing livestock farming.
Criteria 5	Acceptability: Villages must be willing to participate and receive the proposed interventions.

All villages that met the eligibility criteria were listed in Excel by commune or departments of interventions for Burkina Faso and Senegal, respectively. Simple random sampling of eligible villages was then conducted using a random number generator. In Burkina Faso, we also paid particular attention to the challenging and changing security context.

In Senegal, 21 eligible villages were identified and five were selected; Djilor, Simal, Mbam, Mara, and Toune Mandakh (Table 2 and Figure 4).

**Table 2.** Intervention areas selected in Senegal.

Intervention regions	Intervention communes	Selected villages	Population
Fatick	Fimela	Djilor	970
		Simal	2,416
	Mbam	Mbam	3,340
Kaffrine	Diokoul Mbelbouck	Mara	1,232
		Toune Mandakh	1,342

### Map of ELUZO intervention areas in Senegal

In Burkina Faso, two villages in the commune of Tenkodogo, that met eligibility criteria, were selected because of pre-existing agreements established during earlier phases of community engagement. The other villages were randomly chosen, using a random number generator among 127 eligible villages. Additionally, after randomly selecting the village of Secteur 3- Bazin, the neighboring village of Larga was also included, as they form a contiguous settlement making boundaries difficult to define. Thus, for Burkina Faso, seven villages were included in the project (Table 3 and Figure 5).

**Table 3.** Intervention areas selected in Burkina Faso.

Intervention regions	Intervention communes	Selected villages	Population
Centre-Est	Tenkodogo	Soumagou	2,162
		Oueguedo	1,589
Plateau-Central	Zoungou	Silmiougou	1,628
Centre-Sud	Manga	Larga	641
		Secteur 3 – Bazin	10,815
Centre-Ouest	Reo	Bonyolo	5,843
	Nanoro	Poessi	3,441

### Map of ELUZO intervention areas in Burkina Faso

### Baseline assessment

ELUZO aimed to generate a comprehensive One Health overview by collecting baseline data at the human-animal-environmental interface. Baseline data includes all qualitative and quantitative data gathered about each village between June and October 2024, prior to implementation of pilot project interventions. Quantitative data consisted of surveys while qualitative data included individual interviews, focus groups, and participatory walks.

Baseline data collection aimed to obtain information at the intersection of human, animal and environmental health across four themes considered essential for developing the interventions and assessing their effectiveness: (1) Community context and resources: main geographic, socio-economic, and cultural characteristics of each village, socio-demographic characteristics of the community members, and available household resources (human and financial); (2) Livestock and farming practices: animal species raised and associated farming practices, including where the animals are housed, related costs and yields, prevention measures applied; (3) Health and zoonotic risks: main health problems affecting animals, general human health perception and food security, knowledge, attitudes and practices regarding human and/or animal infectious diseases; (4) WASH-related questions : use of latrines and water and sanitation measures in place; and (5) Gender dynamics: women’s involvement in farming activities, resource distribution within households, and role of women in management, revenue control, and decision-making. These data, combined with data from literature reviews helped guide identification and selection of interventions.

All data collection tools were informed by publicly available, validated instruments previously developed for research or development practitioners. For example, WASH-related questions were informed by WHO/UNICEF Joint Monitoring Programme for Water Supply, Sanitation and Hygiene (JMP) [35], food security questions by guidelines from FAO documents [36], and gender-related questions by Women’s Empowerment in Livestock Index (WELI) [37]. Additional questions for surveys and qualitative methods were added based on the expertise and local field experience of project partners. All the tools used (surveys, interview guides, focus group guides, and participatory mapping materials) were co-developed with experts from multiple disciplines and partners institutions, and validated by field partners. They are available in the Open Science Framework (OSF) repository (see supplementary material).

### Baseline quantitative components

In each participating village of Senegal and Burkina Faso, a cross-sectional survey was conducted in June–July 2024 to provide a snapshot of household socio-economic conditions, animal and human health status and concerns, and knowledge, attitudes, and practices related to zoonoses.

#### Sample size for the baseline quantitative component

The sample size was determined using Cochran’s statistical formula (95% confidence level and 5% margin of error) for proportions [38] to ensure adequate representation by age and sex of the target population. To maximize sample sizes, it was assumed that 50% of the population was engaged in farming activities.

For Senegal, the sample size per village was adjusted according to the 2018 census age and sex distribution, with a target of 52% women and 48% men (Table 4). Women and men were defined as being aged 18 and above while girls and boys were between 15 and 17 years old.

**Table 4.** Sample size and distribution of survey participants by age and sex in Senegal.

Commune	Village	Population	Sample size	Total men (18+)	Total women (18+)	Total boys (15–17)	Total girls (15–17)
Fimela	Djilor Simal	3,386 970 2,416	346 104 242	133 40 93	144 43 101	33 10 23	36 11 25
Mbam	Mbam	3,340	345	133	143	33	36
Diokoul Mbelbouck	Mara Toune Mandakh	2,574 1,232 1,342	335 161 174	129 62 67	139 67 72	32 15 17	35 17 18
Total	All	**9,300**	**1026**	**395**	**426**	**98**	**107**

For Burkina Faso, sampling proportions were adjusted using population census data collected prior to the study by local partners, with individuals aged 15–17 years representing approximately 30% of the population and final gender proportions ranging from 51–56% women and 44–49% men, depending on the village (Table 5).

**Table 5.** Sample size and distribution of survey participants by age and sex in Burkina Faso.

Commune	Village	Population	Sample size	Total men (18+)	Total women (18+)	Total boys (15–17)	Total girls (15–17)
Tenkodogo	Soumagou Ouéguédo	3751 2162 1589	349	114	130	49	56
Zoungou	Silmiougou	1628	311	97	121	41	52
Manga	Larga Secteur 3 - Bazin	11456 641 10.815	372	127	134	54	57
Reo	Bonyolo	5.843	362	123	130	53	56
Nanoro	Poessi	3.441	346	106	136	46	58
Total	**All**	**26109**	**1740**	**567**	**651**	**243**	**279**

#### Source, target and study population

The source population consisted of eligible villages (i.e. those meeting the inclusion criteria listed in Table 1) located in the predefined areas of intervention in Burkina Faso and Senegal.

**Figure 6.**
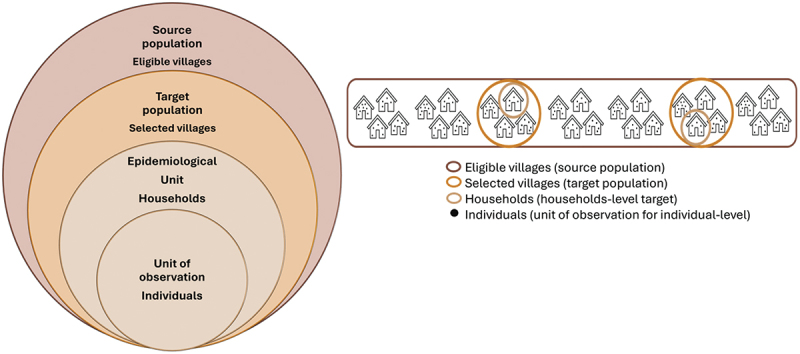
Target, source population, epidemiological unit and individual unit of observation.

The target population was comprised of the selected villages. Within these villages, a census of households engaged in small livestock farming was conducted, from which accessible households (the epidemiological unit) were randomly sampled until the required sample size was reached (see Figure 6).

For outcomes analysed at the individual level, household members or animals owned by the household were considered as the unit of observation.

### Source, target and study population

#### Survey questionnaires

The questionnaire was structured in three sections. The first section included household-level socio-economic and demographic questions that were answered by the head of the household, defined as the person taking the decisions for the household and managing the revenues [4,39]. The second section involved other household members – including women aged 18 years or over, girls and boys 15–17 years, and men 18+ – to investigate household farming activities, animal health concerns, self-rated health perception and specific questions about rabies and avian influenza (see OSF repository for complete materials).

The third section was administered exclusively to women engaged in livestock farming, exploring income generated from their animals, access and use of revenues, and strategies to improve yields and empowerment.

All sections of the questionnaire were validated by field partners to ensure contextual appropriateness. Facilitators, speaking the local dialects (Sérère, Wolof, Pulaar, Diola in Senegal; Mooré, Bissa, Fulfuldé, Gourounsi/liélé in Burkina Faso) were recruited locally to administer the questionnaires in local languages and record responses in French on a Kobo Toolbox. Facilitators received standardized training and supervision to ensure data quality, consistent translation across languages and uniform administration and recording of responses.

The questionnaire was pre-tested with a pilot group in both countries (approximately 30 community members) to ensure question clarity, relevance, and questionnaire completion time.

### Baseline qualitative component

As part of the qualitative component, focus groups, interviews, and participatory mapping exercises were conducted. These methods aim to address themes that could not be fully explored through the survey and to triangulate/validate the quantitative findings.

#### Participants selection

In each participating village, up to four focus groups of six to eight participants aged 18 and above were conducted, resulting in a maximum of 24–32 participants per village. Participants included community members and resource personnel, such as healthcare workers, community leaders and members of women’s associations (Table 6). For the interviews, key informants representing institutions such as NGOs, government ministries, universities, and communities were selected (Table 6).

**Table 6.** Categories of participants included in focus groups and interviews.

Category	Data collection method	Description	Objective
Community members	Focus groups	Individuals living in the selected villages.	Capture household-level practices, experiences, and perceptions.
Resources people in the communities	Focus groups and interviews	Key resource persons such as community leaders, women’s association members and professionals working in the community (e.g. veterinarians, health agents).	Gather additional data on community organization, local animal husbandry, and health practices.
Institutional stakeholders	Interviews	Representatives or experts from ministries, universities, NGOs, and local authorities.	Provide insights on surveillance systems, governance, women’s rights, etc.

Interview participants from the institutions and ministries were selected based on existing relationships with the local partners, while community members participating in focus groups were identified by community leaders and women’s cooperatives (organized groups of women who support one another through income-generating activities such as gardening, textile making, and small-scale livestock rearing).

The final number of focus groups and interviews balanced the need for diversity among participants, scientific rigour and logistical limitations. In total, 12 focus groups and 20 interviews were conducted in Senegal, while 15 focus groups and 14 interviews were done in Burkina Faso.

#### Focus groups

Focus groups were organized separately for women and men, to facilitate open discussion and account for gender dynamics. Women facilitators were specifically chosen for women’s groups. Discussions were conducted in local dialects and recorded for transcription.

Different themes were discussed in different focus groups and for logistical reasons, not all focus group themes were covered in each participating village (Table 7).

**Table 7.** Focus group themes, participant numbers, objectives and villages where it is was conducted.

Themes	Participant numbers	Focus group objective	Participating villages
Gender thematic group #1	6–8 women	Discuss women’s perceptions on gender equality, access to resources, their role in household and decision-making.	Senegal: Mara, Simal, Mbam Burkina Faso: Bonyolo, Soumagou, Larga
Gender thematic group #2	6–8 men	Discuss men’s perceptions on gender equality, women’s access to resources, and women’s role in the household and in decision-making.	Senegal: Mara, Simal, Mbam Burkina Faso: Bonyolo, Soumagou, Larga
Group #3: Zoonoses and animal health	6–8 women	Explore experiences with zoonoses and human and animal health problems, including preventive measures and resources available.	Senegal: Mara, Simal, Mbam Burkina Faso: Bonyolo, Soumagou, Larga, Ouéguédo, Poessi, Silmiougou
Group #4: Farming practices	6–8 women	Collect additional information on farming practices, animal productivity (yields) and how it could be improved.	Senegal: Mara, Simal, Mbam Burkina Faso: Bonyolo, Soumagou, Larga

See supplementary material for the four focus groups guides.

#### Interviews

Interviews were conducted with experts, professionals working within communities and institutional stakeholders to identify major community health problems and related surveillance programs in place in both countries, as well as to gather insights on gender equity issues and any existing interventions or policies addressing these health and equity challenges. Specific topics included livestock practices and their financial aspects, community challenges related to zoonoses, disease control response plans, the role of livestock products in the local food system, and gender dynamics (see supplementary material).

Interviews were conducted one-on-one, either in person or by phone when necessary, and recorded. Institutional representatives have knowledge of policy, governance, and legal aspects related to animal, environmental, and human health, while field agents possess in-depth knowledge of their community.

The same facilitators as for the focus groups conducted the interviews, either in French or in local dialects.

#### Participatory walks and mapping

Participatory mapping is a technique used to show relationships between specific places within communities and how community members perceive or interact with them [40]. Specifically, in ELUZO, participatory mapping was used to identify public places in the participating villages where zoonotic transmission risks could be high (e.g. water points, market areas, slaughter places, public latrines). Men, women, boys and girls participated together in the identification and characterization of key places. In Senegal, the process consisted of four activities: (1) The mapping of the village on large sheets of paper using markers and sticky notes; (2) identification of the top three locations within the community that posed a health risk to humans and (3) animals (by consensus); and (4) participatory walk to observe and document the locations identified in #2 and #3. The mapping process was done in all 5 villages in Senegal and typically involved 20–30 community members. In Burkina Faso, for security reasons, only steps 1–3 were conducted. Mapping activities were carried out in each of the seven selected villages during community assemblies, involving approximately 30 community members.

Activities were conducted by the same facilitators as for the focus groups and interviews. Facilitators documented observations and made pictures of key areas identified. Maps produced in both countries were digitized, and facilitator guides can be found in supplementary material.

### Literature reviews

To complement quantitative and qualitative data collection activities, several reviews were conducted as part of the baseline assessment to enrich our understanding of the local context and identify the gaps.

Three types of reviews were conducted;

- A Scoping review on zoonotic diseases transmitted by livestock animals (small ruminants, pigs and poultry) in Senegal and Burkina Faso: to describe and map circulating zoonoses in both countries and assess reported cases in the participating villages, in line with objective 2 (Preregistration on OSF registries https://osf.io/kjume).
- Narrative reviews on the surveillance systems implemented in both countries. Summaries described surveillance systems in place and how zoonotic events are currently detected and managed at the local and national levels. These reviews relied mainly on grey literature (e.g. governmental reports, technical documents, unpublished field reports).
- A review of policies, regulatory frameworks, and sociocultural norms shaping women’s empowerment in agriculture and livestock in both countries: To provide context on the role and rights of women, their access to resources and participation in income-generating activities relevant for interventions aligned with gender equity goals.

### Baseline analysis and triangulation

For each component of the baseline assessment, specific reports and results were compiled. Quantitative survey data were analysed using descriptive statistics that accounted for the multistage design and clustering at the household and individual levels. Qualitative data (interviews, focus groups, participatory mapping) were analysed through thematic content analysis using QDA Miner [41]. Reports from participative mapping exercises documented field observations related to public places, water points, market areas, waste sites and latrines, supported by photographs and participants notes. Findings from the three types of literature reviews were synthesized separately to provide additional information about women’s rights and access to resources, zoonotic agents in the regions of interventions, and surveillance systems.

To guide the identification of interventions, the data from all sources were triangulated [42–44]. Triangulation consisted of a theme-based comparison of findings across all data sources and participant groups (see Figure 3). A matrix was developed in which methods (i.e. surveys, interviews, focus groups, participatory mapping, and literature reviews) were compared across major thematic area (e.g. water access, livestock management, gender dynamics, zoonotic risks). For every theme-method combination, the main findings were extracted and summarized for all participant groups and discrepancies and complementarities were examined. Particular attention was given to the experiences of women and girls. This mixed method approach allowed us to gather substantial information on humans and animal health, common livestock practices, and household and environmental conditions.

Following triangulation, a problem tree for each country summarizing the main challenges related to human, animal and environmental health was depicted. These challenges were discussed collaboratively among Senegal, Burkina Faso and Canadian team members to inform intervention prioritization.

The triangulation process was conducted between January and March 2025 at the village level independently in Senegal and Burkina Faso to ensure that interventions reflected community-specific priorities. At this stage, descriptive analyses were prioritized to identify key challenges in line with project timelines. Detailed qualitative and quantitative analyses, alongside the results, will be reported in subsequent work.

### Community-level interventions

Triangulation served as the building blocks for identification and design of two types of interventions (Research objective 3): pilot projects and income-generating activities. Pilot projects were defined as medium-scale interventions designed to reduce the impact of zoonotic diseases on women, while promoting health at the human-animal-environment interface. Using participatory methods, selected pilot projects reflect the specific needs of the community, especially those identified by women. Examples of pilot projects include: the construction of latrines or building dedicated areas for animal slaughter or meat inspections. Income-generating activities were designed for women and girls involved in small-scale animal farming or agriculture to favor economic autonomy and empowerment, ideally linked to and supported by the pilot projects. An example included providing training and logistical support to women’s associations to strengthen livestock production and increase revenue.

Selection of interventions (both pilot projects and income-generating activities) and development of implementation strategies were co-constructed with the communities. The process involved participatory discussions during village assemblies, at which community members, particularly women’s cooperatives, and ELUZO representatives could discuss challenges raised by the baseline assessment and possible solutions. Proposed interventions were reviewed by the ELUZO team to assess feasibility, costs, and alignment with project’s objectives, including budgeting and technical consultation with experts. Also, as per GAC requirements, an environmental impact assessment was realized prior to implementation.

The implementation of these interventions was planned for April 2026 to March 2027 and is not detailed in this manuscript as it falls within study results.

#### Capacity building objective

In parallel with the interventions, training and awareness-raising sessions are planned throughout the project duration to strengthen One Health and gender equality, with a focus on women’s roles, decision-making, and self-empowerment.

Awareness activities include radio and theatre, to reach thousands of people in the communities, while training sessions were designed for professionals such as One Health units and members of the One Health platform. One Health units were trained to serve as local trainers to conduct these sessions within communities.

The project also includes experiential learning and training opportunities for students. Canadian trainees support field activities as part of an applied learning experience. Selected students from Senegal and Burkina Faso also benefit from scholarships for master’s or PhD training related to ELUZO.

### Monitoring and evaluation

Monitoring, evaluation and learning (MEL) are integrated throughout all project steps to assess both the implementation of activities and their effects, in alignment with the Theory of Change (Figure 1). Knowledge generated through the baseline assessment, community consultations, implementation activities and follow-up is shared and discussed among community members, local, regional and national leaders and experts, and ELUZO team members to support decision-making. These discussions include collection of additional data about indicators to determine if training sessions, pilot projects and income-generating activities, adoption of prevention measures, or the functioning of One Health units was successful. One Health units also collect other relevant field data, such as major health events, that allow us to track health challenges at the level of the community.

The data collected through MEL support continuous learning for opportunities to improve implementation strategies, help document lessons learned and assess effectiveness of interventions by comparing baseline results to follow-up indicators.

### Ethical considerations

Before starting the project, a study protocol was submitted to the ethics committees of Senegal (Comité National d’Éthique pour la Recherche en Santé - CNERS), Burkina Faso (Comité d’Éthique pour la Recherche en Santé - CERS) and the two Canadian institutions (Comité d’Éthique de la Recherche en Sciences et En Santé - CERSES – of UdeM and research ethic boards – REBs – of University of Guelph). All participants were informed about the purpose of the project and gave consent. Due to low literacy levels and language barriers, consent was usually provided orally to interviewers who ensured participants’ understanding and confirmed consent prior to participation. Written consent was provided by participants who were literate and able to review written study materials. For participants aged 15–18 years, consent was obtained from a parent or legal guardian, along with the participant’s verbal consent. At the time of consent all participants were informed of their right to withdraw from the study at any time or to skip any questions they felt uncomfortable answering. All information provided was anonymized to ensure confidentiality.

The names of the participating villages are reported because ELUZO is a community-based intervention project implemented in collaboration with national and local authorities and participating communities. As such, the intervention sites are publicly known. However, no information allowing the identification of individual participants is given and future publications will report findings in a manner that guaranties participant confidentiality.

## Discussion

While the project results are not presented here, this paper describes the structure and methodological framework of ELUZO and demonstrates how an integrated One Health strategy can be operationalized in rural settings through participatory methods, mixed data collection, and intersectoral collaboration.

Although the same approaches, tools and implementation processes were applied in both countries, results are not expected to be fully comparable across all dimensions given that the villages present distinct socio-economic characteristics and identified different intervention priorities.

Key methodological and operational lessons emerged. First, bringing international development and research methodologies together from the outset required reconciling different timelines, expectations, and objectives. Establishing shared timelines, deliverables and methodological plans from the beginning was essential to bridge different development and research realities. These challenges are consistent with the implementation research literature, where projects need to balance scientific objectives with socio-cultural, operational and logistical realities while engaging multiple stakeholders [45].

Second, co-constructing the project with communities required extensive time investments, frequent field consultations, and cultural and linguistic adaptations. Integrating iterative feedback loops, relying on trained local facilitators, and scheduling consultations to minimize participant burden helped address these challenges. While resource intensive, participatory approaches are recognized as an important mechanism to build trust, understand community priorities and improve the relevance, acceptability and sustainability of interventions [46,47]. The long-term sustainability of such initiatives ultimately depends on the level of ownership felt by the communities themselves, highlighting the central role of capacity building and involvement of community members at every step of the process [46].

Third, adopting a ‘do not harm’ approach [48] was essential to avoid reinforcing vulnerabilities or sensitive gender dynamics. Involving both women and men across all project steps, combined with gender equality training, empowered women and girls and supported more equity across genders while reducing unintended negative effects of interventions.

Finally, interdisciplinary and intersectoral collaboration, integral to One Health projects, required intense coordination and process validation. Although interdisciplinarity and intersectoral One Health approaches are increasingly recognized for their added value in addressing complex health issues [16], they are frequently reported as challenging to implement due to the coordination required, ongoing disciplinary barriers, language barriers, and the need to reconcile diverse stakeholder priorities and objectives [49,50]. In ELUZO, clear definition of roles and responsibilities, and regular communication facilitated shared understandings and efficiency.

## Supplementary Material

Hubert_GHA_Supplementary material_Table S1.docx

## Data Availability

All study tools used in this protocol, including survey questionnaires, interview and focus group guides, and mapping tools, are openly available (in French) on the project’s OSF Registry (https://osf.io/jx7cd). As this article presents a study protocol, no results are currently available.
