# Correction

**DOI:** 10.1080/16549716.2026.2714680

**Published:** 2026-08-13

**Article title**: Operationalizing the One Health approach at the intersection of research and development: a study protocol for the ELUZO project in Senegal and Burkina Faso.

**Authors**: Marine Hubert, Magali Martind, Hélène Carabin, Jane Parmley, Maude Gilbert-Vanassed, Alexis Kafando et al.

**Journal**: *Global Health Action*

**Bibliometrics**: Volume 19, Number 1, 2705653

**DOI**: http://dx.doi.org/10.1080/16549716.2026.2705653

When this article was first published, headings intended as figure titles were inadvertently included as section headings. These have now been removed.

