# Supplementary material for: Operationalizing the One Health approach at the intersection of research and development: a study protocol for the ELUZO project in Senegal and Burkina Faso
Source: Glob Health Action. 2026 Jul 31;19(1):2705653. doi: 10.1080/16549716.2026.2705653 (PMC13431021; doi:10.1080/16549716.2026.2705653)
Supplement: Hubert_GHA_Supplementary material_Table S1.docx [file ZGHA_A_2705653_SM6539.docx]

**Supplementary material**

**Operationalizing the One Health Approach at the Intersection of Research and Development: A study protocol for the ELUZO project in Senegal and Burkina Faso**

***Supplementary Table S1: Partner Institutions and their roles in Canada, Burkina Faso and Senegal for the ELUZO project***

| Country | Partners | Roles | Expertise |
| --- | --- | --- | --- |
| Canada | Université de Montréal [34] | Leading partner; responsible for the overall management and coordination of the project and designated point of contact with GAC. | International development and cooperation, public health, One Health, environmental health, animal health, epidemiology, food security and nutrition. |
|  | University of Guelph [35] | Responsible for providing resources in monitoring and evaluation, animal and environmental health, and WASH. | Zoonoses, Global public health, One Health, environmental health, integrated surveillance systems and water, hygiene, and sanitation. |
| Senegal |  |  |  |
|  | COMI (Cooperazione per il Mondo in Via di Sviluppo) [36] | Country focal point; project management, contact with authorities, coordination of field partners and financial and logistical aspects. | International development and cooperation, sustainable agriculture and livestock development, women’s empowerment and capacity building. |
|  | Cheikh Anta Diop University of Dakar (UCAD) [38] | Responsible for data collection and analysis, monitoring and evaluation, human health expertise, scientific coordination and gender equality activities. | Public health, social sciences. |
|  | Ecole Inter-Etats des Sciences et Médecine Vétérinaires (EISMV) of Dakar [39] | Responsible for data collection and animal health and epidemiology expertise. | Animal Health, epidemiology. |
|  | Adaf Yungar | Responsible for project operations and community mobilization in the Fatick region. | Community training and knowledge transfer, agricultural improvement, sanitation and rural water supply. |
| Burkina Faso |  | |  |
|  | Société d'Études et de Recherche en Santé Publique (SERSAP) [37] | Country focal point; project management, contact with authorities, coordination of field partners and financial and logistical aspects. | Human health, public health, health systems strengthening, capacity building. |
|  | Institut de Recherche en Sciences de la Santé (IRSS) [40] | Responsible for surveys and data collection and human and animal health expertise | Human and animal health |
|  | Université Joseph Ki-Zerbo (UJKZ) [41] | Responsible for training, gender equality, and dissemination of results. | Human health, social sciences, women’s empowerment, capacity building. |
|  | One Health Burkina Faso Association (ASSO-OHBF) | Responsible for scientific coordination and community mobilization and engagement. | One Health promotion. |
|  | Croix-Rouge Burkinabè (CRBF) [42] | Responsible for community mobilization and security monitoring. | Community training and capacity building. |
